# Genomic Insights into Drug Resistance Determinants in *Cedecea neteri*, A Rare Opportunistic Pathogen

**DOI:** 10.3390/microorganisms9081741

**Published:** 2021-08-15

**Authors:** Dorothea K. Thompson, Stephen M. Sharkady

**Affiliations:** Department of Pharmaceutical Sciences, College of Pharmacy & Health Sciences, Campbell University, Buies Creek, NC 27506, USA; sharkady@campbell.edu

**Keywords:** *Cedecea neteri*, opportunistic pathogen, drug-resistance genes, β-lactamases, multidrug efflux pumps, genomic islands

## Abstract

*Cedecea*, a genus in the *Enterobacteriaceae* family, includes several opportunistic pathogens reported to cause an array of sporadic acute infections, most notably of the lung and bloodstream. One species, *Cedecea neteri*, is associated with cases of bacteremia in immunocompromised hosts and has documented resistance to different antibiotics, including β-lactams and colistin. Despite the potential to inflict serious infections, knowledge about drug resistance determinants in *Cedecea* is limited. In this study, we utilized whole-genome sequence data available for three environmental strains (SSMD04, M006, ND14a) of *C. neteri* and various bioinformatics tools to analyze drug resistance genes in this bacterium. All three genomes harbor multiple chromosome-encoded β-lactamase genes. A deeper analysis of β-lactamase genes in SSMD04 revealed four metallo-β-lactamases, a novel variant, and a CMY/ACT-type AmpC putatively regulated by a divergently transcribed AmpR. Homologs of known resistance-nodulation-cell division (RND)-type multidrug efflux pumps such as OqxB, AcrB, AcrD, and MdtBC were also identified. Genomic island prediction for SSMD04 indicated that *tolC*, involved in drug and toxin export across the outer membrane of Gram-negative bacteria, was acquired by a transposase-mediated genetic transfer mechanism. Our study provides new insights into drug resistance mechanisms of an environmental microorganism capable of behaving as a clinically relevant opportunistic pathogen.

## 1. Introduction

The genus *Cedecea* comprises Gram-negative, facultatively anaerobic bacilli that are non-sporulating and fermentative [1]. Like other genera in the *Enterobacteriaceae* family, *Cedecea* species are widely distributed in aquatic and soil environments, as well as associated with plants, insects, the human gut microbiome, and non-human animals (reviewed in [2,3]). Three validly recognized species (*Cedecea davisae*, *Cedecea lapagei*, and *Cedecea neteri*) have documented clinical relevance in humans and collectively, have been reported to cause such diverse acute infections as pneumonia [4,5,6,7,8,9], bacteremia [8,9,10,11,12,13,14,15,16,17,18,19], cutaneous and oral ulcers [13,14,20,21], and dialysis-related peritonitis [22]. Infections attributed to *Cedecea* occur predominantly in severely immunocompromised hosts, underscoring the opportunistic nature of its pathogenicity.

While *Cedecea* infections have been reported sporadically in the literature, clinical cases of this emerging opportunistic human pathogen continue to occur worldwide. Clinical strains of *Cedecea* species exhibit natural resistance to various antimicrobial agents, including ampicillin, ampicillin-sulbactam, cefazolin, cephalothin, cefoxitin, and colistin [3]. The multidrug-resistant phenotype of one *C. lapagei* isolate was attributed, in part, to the acquisition of the *bla*_NDM-1_ gene, which encodes the New Delhi metallo-β-lactamase-1 (NDM-1) [18]. This clinical isolate was resistant to carbapenems, monobactams, and extended-spectrum cephalosporins. Recently, additional *bla*_NDM-1_-harboring *Cedecea* isolates have been reported [23,24]. The dissemination of NDM-1 is of serious public health concern as this metallo-β-lactamase severely limits the chemotherapeutic options available for treating bacterial infections. Apart from the detection of β-lactamase expression in certain *Cedecea* strains [18,25], knowledge on the genetic basis of drug resistance phenotypes in this genus is limited. The pathogenic potential of *Cedecea* species is likely under-recognized due to its low reported frequency of association with human infections and the fact that these organisms are not linked to specific disease states but, instead, cause a wide spectrum of acute infections in compromised hosts.

Opportunistic *C. neteri* infections typically present as bacteremia [10,12], although recently *C. neteri* was identified as the etiological agent of a urinary tract infection in a pregnant female with polyhydramnios [26]. One of the rare fatal patient outcomes associated with clinical isolates of *Cedecea* spp. involved a *C. neteri* infection in the bloodstream of an individual with systemic lupus erythematosus (SLE) [12]. Given the potentially serious nature of these opportunistic infections, research is needed on identifying the genes implicated in drug resistance phenotypes that impact treatment of *Cedecea*. In this study, we focused our attention on *C. neteri* because whole-genome sequence (WGS) information is available for multiple strains of this species. WGS data for three environmental strains of *C. neteri* and different bioinformatics tools were utilized to provide insight into the predicted drug resistance determinants of this species. Only a draft genome at the unassembled contig level is available for the type strain *C. neteri* ATCC 33855 [10], isolated from a human foot, so we focused our queries on the completed genome sequence of *C. neteri* SSMD04 [27] and compared results to the genomes of *C. neteri* M006 [28] and ND14a. Our findings highlight the value of genome-based explorations in contributing to our understanding of antimicrobial resistance in little studied environmental microorganisms with the capability of behaving as clinically relevant opportunistic pathogens.

## 2. Materials and Methods

### 2.1. Bacterial Genomes 

Detailed information about the bacterial genomes analyzed in this study are presented in Table 1. Completely sequenced and annotated genomes for the following strains have been previously deposited in GenBank: *C. neteri* SSMD04 (GenBank accession no. CP009451.1), *C. neteri* M006 (GenBank accession no. CP009458.1), *C. neteri* ND14a (GenBank accession no. CP009459.1), and *Klebsiella michiganensis* RC10 (GenBank accession no. CP011077.1). These genomes do not contain any extrachromosomal elements. SSMD04 was used as the query strain in sequence homology searches and comparative genomic analyses.

### 2.2. Sequence Database Search and Bioinformatics Tools

Initial searches based on gene and protein annotation were performed against the KEGG (Kyoto Encyclopedia of Gene and Genomes) integrated database resource (https://www.genome.jp/kegg/, accessed on 14 August 2021; [29]) using “beta-lactamase”, “transporter”, “efflux pump”, “antibiotic resistance”, “drug resistance”, and “multidrug resistance” as search terms. Similarity of intra- and interspecies protein coding sequences (CDSs) was computed using BLASTP [30] with a default E-value threshold of 10.0 and the BLOSUM62 amino-acid scoring matrix. For all BLASTP analyses, the *C. neteri* SSMD04 CDS represented the query sequence. ClustalW [31] was used to construct multiple alignments of protein sequences. Sequence signatures or motifs specific for the different Ambler classes of β-lactamases were identified by a combination of manual scrutiny and multiple sequence alignment. A phylogram (midpoint-rooted tree) was generated using the phylogenetic analysis pipeline of the Environment for Tree Exploration, version 3.1.1 (ETE3) [32] and FastTree v2.1.8 [33] with default parameters.

### 2.3. Comparative Genomic Analysis

Average nucleotide and amino acid identities between *C. neteri* SSMD04 and *Klebsiella michiganensis* RC10 were determined using the online tools found at http://enve-omics.ce.gatech.edu/ani/ (accessed on 14 August 2021) and http://enve-omics.ce.gatech.edu/aai/ (accessed on 14 August 2021), respectively [34]. Genomic islands (GIs) in *C. neteri* SSMD04 and *Klebsiella michiganensis* RC10 were predicted and visualized using IslandViewer 4 (https://www.pathogenomics.sfu.ca/islandviewer/, accessed on 14 August 2021), a webserver that integrates four independent GI prediction methods: IslandPath-DIMOB, SIGI-HMM, IslandPick, and Islander [35]. *Klebsiella michiganensis* RC10 was selected for genomic comparison because this environmental bacterium shares a similar opportunistic pathogenesis as *C. neteri* and exhibits high sequence identity with *C. neteri* at the individual β-lactamase gene level (see Table 2). Furthermore, IslandPick in the IslandViewer 4 webserver identified the RC10 genome as a suitable comparison genome based on phylogeny for prediction of the most probable GIs in the *C. neteri* SSMD04 genome.

## 3. Results and Discussion

### 3.1. Multiple Metallo-β-Lactamase-Encoding Genes in C. neteri

Six open-reading frames (ORFs) in the *C. neteri* SSMD04 genome (Cnt) are annotated as unclassified β-lactamases based on a previous KEGG database search [36], compared with 9 ORFs in strain M006 and 11 ORFs in strain ND14a. As environmental *Cedecea* species exhibit a natural phenotype of resistance to certain clinically important β-lactam antibiotics [3], we further analyzed these six putative β-lactamase-encoding ORFs in more detail. The deduced primary sequences of four of these genes have the metallo-beta-lactamase (MBL) protein fold (αβ/αβ) distinctive of the metallo-hydrolase/oxidoreductase superfamily based on the presence of Pfam domains (Table 2). Bacterial MBLs are clinically relevant because these enzymes hydrolyze and inactivate a broad spectrum of therapeutically important β-lactam antibiotics, including carbapenems, and are refractory to inhibition by clavulanate, sulbactam, and tazobactam [37]. MBLs require a divalent metal ion, usually zinc, as a cofactor for β-lactam hydrolysis (reviewed in [38]). While Class B metallo-β-lactamases exhibit substantial divergence in terms of molecular structure and function, sequence analysis revealed that three (Cnt00700, Cnt16535, and Cnt22070) of the four putative *C. neteri* SSMD04 MBLs contained the strictly conserved metal-binding motif, H-X-H-X-D-H, which is involved in specific Zn^2+^ interactions and functions as the MBL active-site center (Table 2; [39,40]). The other predicted metallo-β-lactamase, Cnt03975, lacked the conserved group B-specific motif involved in zinc ion coordination, but harbored conserved histidine residues at positions 113, 186, and 220, and two aspartic acid residues at positions 85 and 87 which may contribute to activity.

**Table 2:** Predicted β-lactamase-encoding genes in *C. neteri* SSMD04.

Gene Locus (cnt)	BLASTP Search Bacterium (% AA Sequence Identity)	Predicted Gene Product	Ambler Class	Predicted Subclass/Family Based on Sequence Fingerprints	Group-Specific Signatures/Conserved Residues
00700	kmi (96), cem (95) cen (95), clap (91)	Metallo-β-lactamase	B	B3/L1 * ^198^P-G-H-T-P-G^203^	^129^H-x-H-x-D-H^134^ D-97, L-112, G-203, H-224
03975	cem (95), cen (95) kmi (95), clap (93)	Metallo-β-lactamase	B	NI	Lacks H-x-H-x-D-H motif D-85, D-87, H-113, H-186, H-220, G-228
10470	cen (94), kmi (93) cem (93), clap (92) ear (76), enc (75)	AmpC (*bla*_CMY/ACT_)	C	CMY ^†^/ACT ^279^R-Y-W-R-(v)-G-(s)-M-Y-Q^288^	^85^S-x-S-K^88^ ^171^Y-A-N^173^ ^238^D-A-E-A^241^ ^308^S-D-N-K^311^ ^336^K-T-G^338^
16535	cem (94), cen (94) kmi (93), clap (93)	Metallo-β-lactamase	B	NI	^156^H-x-H-x-D-H^161^ H-46, D-87, L-101, H-103, G-191, H-186, H-221
22070	cen (90), cem (89) kmi (89), clap (89) ctu (69)	Metallo-β-lactamase	B	NI	^131^H-x-H-x-D-H^136^ H-78, D-93, H-195, H-262, H-263
22350	clap (93), opo (88) hav (87), kas (66), enc (65)	β-lactamase	Putative Class C	Novel variant	^144^S-x-x-K^147^

Abbreviations: AA, amino acid; cnt, *Cedecea neteri* SSMD04; cem, *Cedecea neteri* M006; cen, *Cedecea neteri* ND14a; clap, *Cedecea lapagei* NCTC11466; ctu, *Cronobacter turicensis*; ear, *Klebsiella aerogenes* EA1509E; kas, *Kluyvera ascorbata*; enc, *Enterobacter cloacae* subsp. cloacae ATCC 13047; hav, *Hafnia alvei*; kmi, *Klebsiella michiganensis* RC10; opo, *Obesumbacterium proteus*; NI, not identified. * Subclass of metallo-β-lactamases, as identified by the presence of sequence fingerprint P-G-H-T-P-G [41]. ^†^ CMY family of AmpC β-lactamases, as identified by the presence of sequence fingerprint R-Y-W-R-v-G-s-M-Y-Q [41].

Family-specific motifs can serve as structural ‘fingerprints’ for the assignment of new members to β-lactamase families, and the three subclasses of MBLs (B1, B2, and B3) possess distinct conserved sets of amino acid residues [41]. We searched the amino acid sequences of the four putative *C. neteri* MBLs for the presence of molecular fingerprints and identified the sequence ^198^P-G-H-T-P-G^203^ in a fingerprint (HFMPGHTPGS) previously identified as characterizing subclass B3 MBLs [41]. Of the four SSMD04 ORFs with a predicted MBL protein fold, the PGHTPG motif was only identified in Cnt00700, suggesting that this MBL may belong to subclass B3 (Table 2). The motif ^195^V-P-L-P-G-H-T-P-G-H^204^ is conserved in predicted Class B β-lactamase sequences from *C. neteri* strains (SSMD04, M006, ND14a), *C. lapagei* NCTC11466, and *Klebsiella michiganensis* RC10. Interestingly, subclass B3 MBLs have been identified in the genus *Chryseobacterium*, whose members inhabit environmental niches and exhibit the capacity to behave as opportunistic pathogens [42]. Cnt00700 and the subclass B3-metallo-β-lactamase (CPS-1) from *Chryseobacterium piscium* share the conserved sequence motif PGHTxG, which was not found in the other putative MBLs encoded in the SSMD04 genome. CPS-1 displayed broad-spectrum hydrolyzing activity against penicillins, cephalothin, some oxyimino-cephalosporins (cefuroxime, ceftriaxone, cefotaxime), cefoxitin, and carbapenems (imipenem, meropenem, doripenem) [43].

### 3.2. A Novel β-Lactamase Gene in the SSMD04 Genome

Most of the genes with predicted functions in β-lactamase-mediated antibiotic resistance in *C. neteri* SSMD04 were also conserved in the genomes of *C. neteri* strains M006 and ND14a. The exception was *cnt22350* (ORF JT31_22350), which exhibited 93% amino acid identity to a putative 6-aminohexanoate-dimer hydrolase (NylB) from *C. lapagei* NCTC11466 but had no orthologs in C. neteri strains M006 and ND14a and in *K. michiganensis* (Table 2). The protein product of *cnt22350* displayed 87% amino acid identity with an unclassified β-lactamase from *Hafnia alvei*, a member of the *Enterobacteriaceae* family associated with infrequent diarrheal cases in humans, as well as nosocomial systemic infections [44,45]. BLASTP analysis of Cnt22350 against the KEGG database demonstrated notable amino acid sequence identity to AmpC from *Kluyvera ascorbata* and *Enterobacter cloacae* subsp. *cloacae* ATCC 13047 (66 and 65%, respectively). Multiple sequence alignment further revealed that Cnt22350 lacked most of the known conserved structural elements found in Ambler Class A, C, and D β-lactamases. The conserved ^70^S-X-X-K^73^ motif in Class A and D families was replaced by S-Y-E-G, and the ^64^S-X-X-K^67^ sequence element characterizing Class C β-lactamases was replaced by ^65^R-N-D-Y-R^69^. The sequence ^144^S-V-G-K^147^ was identified within a larger element ^142^S-R-S-V-G-K-S-V-V-S-T-L-V-G^155^ that was highly conserved among homologs and may constitute a novel serine-containing active-site signature. We suggest that Cnt22350 represents a variant β-lactamase that may possess some novel functional features.

### 3.3. CMY/ACT-Type AmpC β-Lactamase 

**Figure 1:**
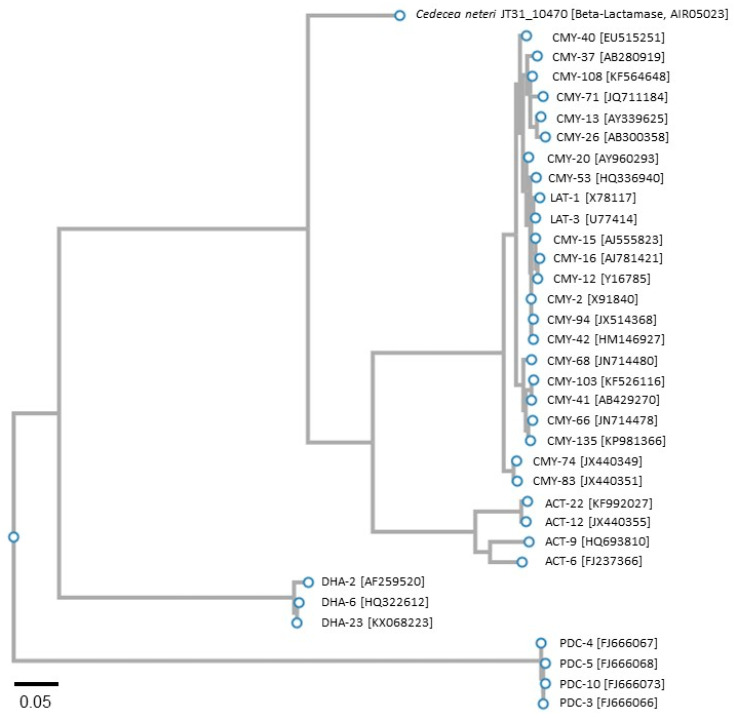
Evolutionary relationships between *C. neteri* JT31_10470 (predicted β-lactamase) and AmpC family variants estimated using the ETE3 phylogenetic analysis pipeline to construct a FastTree v2.1.8 phylogram from top-scoring amino acid alignments. GenBank accession numbers for each protein are provided in brackets.

Previously, we proposed that cnt10470 (ORF JT31_10470) in the *C. neteri* SSMD04 genome encodes an Ambler Class C (AmpC) β-lactamase (cephalosporinase) based on the presence of signature motifs characteristic of Class C active-site serine β-lactamases and a functionally verified *Cedecea davisae* AmpC [25,36]. Various families have been described for AmpC β-lactamases according to polymorphisms in amino acid sequence and include CMY (currently the largest family), ACC, ACT, FOX, LAT, MIR, MOX, and DHA, as well as others (reviewed in [46,47]). AmpC-type variants with expanded-spectrum activity toward imipenem also have been described in *Pseudomonas aeruginosa* and are collectively designated as PDC [48]. To investigate the evolutionary relationship between Cnt10470 and various AmpC clones, a phylogram was constructed using the amino acid sequences from plasmid and chromosomal AmpCs belonging to the families CMY, ACT, LAT, DHA, and PDC based on the top BLASTP hits (Figure 1). The predicted *C. neteri* AmpC is more related to polymorphic CMY- and ACT-type AmpCs compared with DHA- and PDC-type variants. Further sequence analysis indicated that the chromosomal-borne SSMD04 AmpC likely belongs to the CMY/ACT family based on the presence of the sequence fingerprint RYWR(v)G(s)MYQ [41] at amino acid positions 279 to 288 (Table 2). This motif was conserved among Cnt10470 and various CMY and ACT clones, except at positions 283 and 285; however, the same sequence motif was absent in DHA and PDC representatives, which diverged from a less recent common ancestor. The phylogenetic results suggest that the SSMD04 AmpC represents yet another variant in the highly polymorphic CMY/ACT families.

Recent preliminary kinetic studies conducted by this research group demonstrated that purified polyhistidine-tagged versions of the predicted *C. neteri* AmpC from the SSMD04 environmental strain and a clinical isolate, ATCC 33855, readily hydrolyzed chromogenic cephalosporin substrates CENTA [49] and nitrocefin, demonstrating that both recombinant AmpC proteins are functional (data not shown). While CMY variants can exhibit functional differences, the hydrolysis activity profile of CMY-type AmpC enzymes includes penicillins, cephalosporins, cephamycins (e.g., cefoxitin, cefotetan), oxyimino-cephalosporins (e.g., ceftazidime, cefotaxime, ceftriaxone), and monobactams such as aztreonam. In-depth biochemical characterization of the recombinant AmpCs from *C. neteri* SSMD04 and *C. neteri* ATCC 33855 is ongoing.

### 3.4. Genetic Environment of AmpC Gene in C. neteri SSMD04 

**Figure 2:**
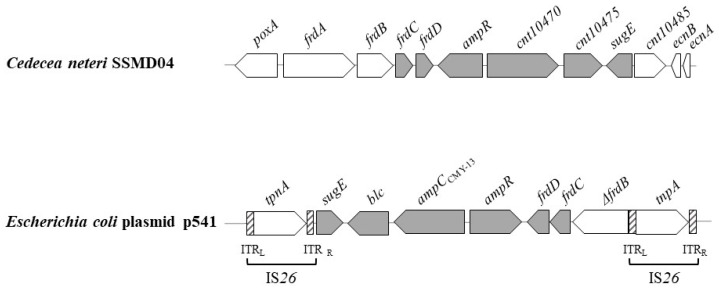
Genetic environment of the JT31_10470 (*cnt10470*) locus on the *C. neteri* SSMD04 chromosome compared to the *ampC*_CMY-13_ locus on the *E. coli* plasmid p541 [50]. Shared genes with similar annotated functions are displayed in gray.

As shown in Figure 2, the genomic organization of the *C. neteri ampC* (*cnt10470*) locus closely resembles the structure of plasmid-mediated *E. coli* CMY-13-type *ampC*, which was originally derived from *Citrobacter freundii* via a transposition-mediated genetic transfer mechanism [50]. The C. neteri AmpC shares 73% amino acid identity to CMY-13. Like the *E. coli* CMY-13 gene, *C. neteri ampC* is linked to a divergently oriented gene annotated as a LysR family transcriptional regulator (Cnt10465), which we previously proposed is AmpR [36]. The Cnt10465 gene product shares high amino acid identity to AmpR from *Cedecea lapagei* (94%), *Enterobacter cloacae* ATCC 13047 (82%), *Kluyvera intermedia* (81%), *Enterobacter ludwigii* (80%), and *Pseudomonas aeruginosa* (64%). The N-terminal helix-turn-helix (HTH) motif in the predicted SSMD04 AmpR contains conserved residues S-38 and K-42 which have been shown to be critical for the DNA-binding activity of AmpR in *P. aeruginosa* [51]. The predicted *C. neteri* AmpR also contains conserved residues G-102 and D-135, which play important structural roles in *P. aeruginosa* AmpR.

The *E. coli* and *C. neteri ampR*-*ampC* loci are flanked by fumarate reductase genes and sugE, which encodes an efflux pump of the small multidrug resistance family (SMR) that confers resistance to quaternary ammonium compounds (Figure 2; [50,52]). A similar genetic organization was identified in the genomes of *C. neteri* M006 and ND14a. The fumarate reductase cluster *frdABCD* is located immediately downstream of SSMD04 *ampR*, and an outer membrane lipoprotein gene, cnt10475, is immediately downstream of *ampC*. The deduced amino acid sequence of Cnt10475 is 76% identical to Blc (apolipoprotein D/lipocalin family protein) from *Salmonella enterica* and *E. coli*.

The presence of a divergently transcribed AmpR homolog suggests that *C. neteri* exhibits an inducible AmpC phenotype in the presence of β-lactam antibiotics. This chromosomal β-lactamase induction mechanism has previously been shown to involve three additional major gene products connected to the peptidoglycan recycling pathway: AmpD (a cytosolic N-acetyl-anhydromuramyl-L-alanine amidase), AmpE (specific function unknown), and AmpG (inner membrane permease responsible for muropeptide transport) ([53]; reviewed in [54]). The SSMD04 chromosome harbors the following genes comprising the specific transcriptional regulatory system for *ampC* expression: *ampD* (*cnt08695*, amidase), *ampE* (*cnt08690*, regulatory protein), and *ampG* (*cnt07450*, muropeptide transporter). The putative *ampD* and *ampE* loci are closely linked on the SSMD04 chromosome and may constitute an *ampDE* operon, the same gene organization that has been reported in other enterobacteria [55,56].

### 3.5. Predicted Multidrug Efflux Pumps in C. neteri 

A ubiquitous microbial mechanism of resistance is drug extrusion via integral membrane transporters, which reduces exposure of the bacterial target to active concentrations of the drug. Five different transporter families confer clinical resistance to various antibiotic classes [57]: ATP-binding cassette (ABC), multidrug and toxic compound extrusion (MATE), major facilitator superfamily (MFS), resistance-nodulation-division (RND), and small multidrug resistance (SMR). Database searches revealed the presence of at least 34, 32, and 33 annotated efflux pump or transporter genes in *C. neteri* strains SSMD04, M006, and ND14a, respectively. Classifications of these predicted efflux pump/transporter genes were distributed across the five different families (ABC, MATE, MFS, RND, and SMR). Efflux pumps of the RND family represent some of the most clinically significant transporter proteins in Gram-negative bacteria because of their broad substrate specificity and association with multidrug resistance (MDR) [58]. Genomes for all three *C. neteri* strains encoded several RND MDR transporters generally annotated as efflux pumps.

**Figure 3:**
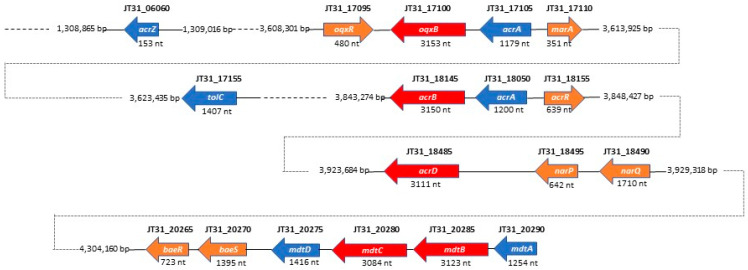
Schematic representation showing the position and size of predicted RND family efflux pump systems and associated genes in the *C. neteri* SSMD04 genome. RND efflux pumps are displayed in red, structural components in blue, and regulatory genes in orange.

By performing in-depth homology searches against the KEGG database, we found homologs of the RND MDR efflux systems AcrAB-TolC, AcrD, OqxAB, and MdtABCD in the SSMD04 genome (Figure 3). BLAST searches indicated that one predicted RND efflux system resembled both OqxAB and AcrAB. Cnt17100 (ORF JT31_17100) has 95% identity to OqxB in *Enterobacter cloacae* and 84% identity to AcrB in *Xanthomonas citri* at the amino acid level. Both OqxB and AcrB are RND transporters that share a consistent transmembrane helical structure [59]. Immediately upstream of JT31_17100 is a gene with 88% amino acid identity to AcrA in *E. cloacae* and 85% identity to OqxA in *Klebsiella variicola*. OqxA and AcrA function as membrane fusion proteins (MFPs) in the RND MDR efflux system. Upstream of the *oqxAB*/*acrAB* gene cluster is a gene (JT31_17110) encoding an AraC family transcriptional regulator that is transcribed divergently. The deduced gene product shares 84% amino acid identity to the transcriptional activator MarA in *Enterobacter ludwigii* and 78% identity to RarA in *Klebsiella pneumoniae*. A second putative regulatory gene (JT31_17095), encoding a Rrf2-type regulator, is located immediately downstream of the *oqxAB*/*acrAB* gene cluster and may be involved in repressing transcription of the putative RND MDR efflux system based on its homology to OqxR. Substrates of the AcrAB efflux pump include cationic dyes (acriflavine), detergents, and antibiotics such as penicillins, cephalosporins, fluoroquinolones, macrolides, chloramphenicol, and tetracycline [60]. OqxAB confers resistance to multiple antimicrobial agents (quinoxalines, quinolones, tigecycline, nitrofurantoin, and chloramphenicol), as well as detergents and disinfectants [59]. The function of the *E. coli* AcrB transporter is dependent on TolC, a multifunctional outer membrane channel [61,62], and a homologous gene with 75% amino acid sequence identity to TolC in *Salmonella enterica* was identified upstream of the *C. neteri acrB*/*oqxB* gene (JT31_17100), suggesting a similar tripartite system in this species (Figure 3). Additionally, the small adaptor protein, AcrZ, which interacts with the AcrAB efflux pump, was identified some distance downstream of the *acrB* gene in a similar genetic organization as the *E. coli* cluster [63]. The AcrZ protein was shown to aid in the binding and export of chloramphenicol and tetracycline by the AcrAB efflux pump, thus enhancing the drug resistance phenotype of *E. coli* [64].

In our analysis, a BLAST search suggested that *C. neteri* JT31_18485 is a homolog of *E. coli* AcrD, an aminoglycoside efflux pump from the RND family [65]. The JT31_18485 gene product showed 87% identity in amino acid sequence to AcrD from *E. coli* and *Shigella sonnei*. In addition, homologous genes encoding the MdtABCD efflux pump system were identified on the *C. neteri* SSMD04 chromosome (Figure 3). The *C. neteri mdtABCD* locus encodes a putative membrane fusion protein (*mdtA*), two RND-type transporters (*mdtB* and *mdtC*), and an MFS-type transporter (*mdtD*). The predicted MdtB and MdtC transmembrane exporter subunits in *C. neteri* exhibited high amino acid sequence identity (89% and 90%, respectively) to their counterparts in *E. cloacae* and *E. coli*. MdtABCD has previously been shown to comprise a multidrug efflux system that confers resistance to novobiocin and deoxycholate [66,67]. The genes *baeS* and *baeR*, which encode a two-component signal transduction system, were identified immediately downstream of the *C. neteri mdtABCD* locus in a similar genetic organization as described for *E. coli*. The BaeSR two-component system positively regulates drug resistance in *E. coli* via the MdtABCD multidrug efflux system [67,68], suggesting that the predicted *mdtABCD* locus in *C. neteri* may be under the transcriptional control of the BaeR response regulator. These putative RND efflux pump systems likely contribute to the intrinsic antimicrobial drug resistance reported for clinical isolates of *C. neteri*. However, the functional role of these homologs of RND efflux pumps and the identity of the specific drug substrates of each pump remain to be established.

A KEGG database search also revealed two pairs of linked genes annotated as *emrA*-like and *emrB*-like MDR transporters of the MFS family on the chromosomes of all three *C. neteri* strains. In *C. neteri* SSMD04, these *emrA*/*emrB*-like gene pairs are JT31_07770/JT31_07775 and JT31_16920/JT31_16915. Multiple sequence alignment showed that the JT31_16920/JT31_16915 pair had the highest sequence identity with the known EmrAB counterpart in *Klebsiella pneumoniae*, *Salmonella enterica*, and *E. coli*. The *emrAB* locus in *E. coli* encodes a MDR pump involved in the extrusion of chemically unrelated antimicrobial agents, including the antibiotics nalidixic acid and thiolactomycin [69]. The putative SSMD04 *emrB*, which encodes a MFS-type efflux pump, shared 93% amino acid sequence identity with *K. pneumoniae* EmrB, 92% identity with *E. coli* EmrB, and 90% identity with *S. enterica* EmrB. The putative *emrA*, which encodes a membrane fusion protein, shared high amino acid sequence identity (82%-86%) with EmrA in *K. pneumoniae*, *E. coli*, and *S. enterica*. In addition, a gene (JT31_16925) annotated as *mprA* is located adjacent to *emrA*. Previous research demonstrated that *mprA* (renamed *emrR*) is part of the *emrAB* operon and functions to repress transcription of *emrAB* [70]. A recent study showed that the EmrAB pump system contributes to colistin resistance in the nosocomial pathogen *Acinetobacter baumannii* [71]. Colistin resistance has been noted as one of the defining properties characterizing *Cedecea* species and is a trait shared by established opportunistic pathogens in the genus *Serratia* [1,10], but the specific mechanism conferring this resistance in *Cedecea* is not known. The possibility that the predicted MFS-type EmrAB efflux pump system may be responsible, at least in part, for the colistin resistance phenotype in *Cedecea* should be explored further, particularly since these species are gaining increased recognition as opportunistic pathogens in the clinical setting.

### 3.6. Comparison of Predicted Genomic Islands in C. neteri and K. michiganensis 

Comparative sequence analysis of chromosome-encoded β-lactamase genes in *C. neteri* SSMD04 revealed a high degree of amino acid sequence identity (89-96%) to orthologous genes in *K. michiganensis* RC10 (Table 2). *K. michiganensis* is an emerging human pathogen that was recently identified as the causative agent of bacteremia in a neutropenic patient with acute myeloid leukemia [72]. Clinical cases of *C. neteri* are markedly similar to the reported *K. michiganensis* case in terms of clinical presentation and the opportunistic nature of infection. Therefore, we performed a whole-genome comparison of *C. neteri* SSMD04 and *K. michiganensis* RC10 to further explore the genetic similarity of these two organisms. The SSMD04 and RC10 genomes share a high average pairwise amino acid identity of 95%, whereas the average pairwise nucleotide identity of 88.8% indicates greater interspecies genetic divergence at the nucleotide level.

**Figure 4:**
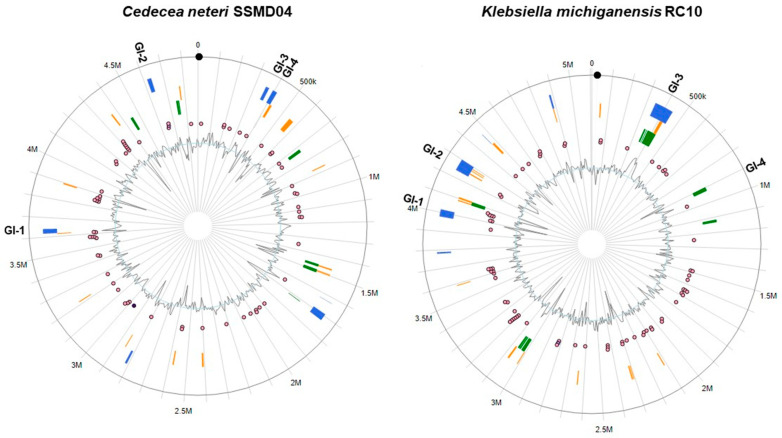
Predicted genomic islands (GIs) and antimicrobial resistance genes in the sequenced genomes of *Cedecea neteri* SSMD04 (GenBank accession number NZ_CP009451.1) and *Klebsiella michiganensis* RC10 (accession number NZ_CP011077.1). Solid rectangles denote GIs predicted by IslandPath-DIMOB (blue), SIGI-HMM (orange), and IslandPick (green) methods using the webserver IslandViewer 4 [35]. Selected genomic islands are labeled GI-1 through GI-4. Predicted AMR genes (pink circles) are also shown.

IslandViewer 4 [35] was utilized to predict GIs present in the genomes of *C. neteri* and *K. michiganensis*, as well as homologs of antimicrobial resistance genes. GIs represent gene regions that originated through probable horizontal gene transfer mechanisms and disproportionately encode for phenotypes that confer enhanced adaptability or competitiveness to microorganisms [35]. Acquired phenotypes include antimicrobial resistance, metal resistance, pathogenicity, as well as phenotypes that contribute to intraspecies diversity and ecological adaptations. In our analysis, two predicted GIs (denoted as GI-1 in Figure 4), one in the *C. neteri* genome and the other in the *K. michiganensis* genome, were strikingly similar in terms of the annotations of clustered genes (Table 3), indicating a probable horizontal transfer event between these bacteria or with a common source. These GIs have not been characterized previously. Notably, several annotated genes clustering on both GI-1s are associated with drug resistance and virulence (Table 3). The first gene, *hlyD*, encodes a membrane fusion protein that is a component of the type 1 secretion machinery responsible for secretion of hemolytic toxin HlyA in uropathogenic *E. coli* strains [73]. The second gene encodes a type I secretion system (T1SS) permease/ATPase and is involved in the secretion of various factors to the extracellular space for biofilm formation and host invasion [74]. Both GI-1s also shared a putative *tolC*, which encodes a multifunctional outer membrane channel. *E. coli* TolC interacts with various inner membrane transporters (e.g., AcrAB, AcrD, and MdtABC) and forms part of the HlyA T1SS complex (reviewed in [75]), thereby playing critical mechanistic roles in the excretion of a wide range of molecules, including antibiotics [76], bile salts [77,78], antimicrobial peptides such as colicin V [79], and the toxin, α-hemolysin [80,81]. Inactivation of *tolC* has been shown to result in bacterial susceptibility to various antibiotics [82]. The *C. neteri* and *K. michiganensis* GI-associated *tolC* shared 96% sequence identity at the amino acid level. Both GI-1s harbored genes encoding transposases and integrases, suggesting that *tolC* was acquired by horizontal means involving genetic transposition.

Interestingly, the *tolC*-harboring GI-1s in the *C. neteri* and *K. michiganensis* chromosomes reside in the same genomic region and in close proximity to genes encoding the RND efflux pump system AcrAB/OqxAB and the multidrug resistance efflux complex, EmrAB, both of which have been described previously in this report. Like the AcrAB efflux pump, the *E. coli* EmrAB pump forms a tripartite efflux system with the outer membrane channel TolC, with the membrane fusion EmrA protein directly interacting with TolC to form an extended periplasmic canal [83]. A gene encoding EmrR, a MarR family-type transcriptional repressor, was also found adjacent to the *emrAB* operon in the SSMD04 and RC10 genomes. In general, homologous genes encoding previously characterized antimicrobial resistance (AMR) genes were not found within predicted GIs, suggesting that known AMR genes in *C. neteri* SSMD04 and *K. michiganensis* RC10 comprise part of the intrinsic drug resistance mechanisms in these organisms. Putative AMR genes were distributed across the entire SSMD04 and RC10 genomes (Figure 4).

Other GIs predicted for *C. neteri* and *K. michiganensis* contained phage proteins, transposon-related proteins, insertion sequences, and numerous hypothetical proteins. Genomic islands 2 and 4 in *C. neteri* SSMD04 contained largely phage-associated proteins and hypothetical proteins (Figure 4). Seven arsenical resistance-related genes were identified on GI-2 in *K. michiganensis* RC10 and included an arsenical efflux pump system, a transcriptional repressor (ArsD) of the arsenical resistance operon, and arsenate reductase, as well as 23 hypothetical proteins. A similar metal resistance GI was not identified in *C. neteri* SSMD04. The RC10 GI-2 also contained an unknown toxin and antitoxin, as well as a MFS transporter gene (VW41_RS19820, WP_045783884.1). Furthermore, GI-2 in *K. michiganensis* was located on the chromosome in close proximity to an unknown MFS transporter gene (VW41_RS19520, WP_045783865.1). GI-3 in the RC10 genome contained genes encoding numerous hypothetical proteins as well as unknown transcriptional regulators, conjugal transfer protein TraG, and multiple integrating conjugative element proteins. 

Comparative GI analysis revealed a gene encoding the virulence factor SrfB on GI-3 in *C. neteri* SSMD04 (locus JT31_RS01505, GenBank accession no. WP_038472517.1) and GI-4 in *K. michiganensis* RC10 (locus VW41_RS04305, GenBank accession no. WP_045781468.1). The putative *srfB* (JT31_01560) on SSMD04 GI-3 is immediately flanked by two ORFs annotated as hypothetical proteins. BLAST searches and multiple sequence alignments suggest that the two hypothetical ORFs linked with *srfB* may represent *srfA* (JT31_01555) and *srfC* (JT31_01565). ORF JT31_01555 showed 53% identity at the deduced amino acid level with *S. enterica* SrfA, and JT31_01565 showed 41% identity with *S. enterica* SrfC. Initially identified in *S. enterica* [84], the *srfABC* operon encodes a tripartite toxin that was shown to exhibit injectable insecticidal activity [85]. Recently, Sun et al. [86] demonstrated that each component of the SrfABC toxin is capable of independently inducing varying degrees of cytotoxicity and apoptosis in human cervical carcinoma cells, while all three proteins are required for full cytotoxicity. The presence of *srfB* on a predicted GI in *C. neteri* and *K. michiganensis* suggests its acquisition via a horizontal gene transfer mechanism and raises the question of whether the product of *srfB* contributes to the opportunistic pathogenicity of these organisms.

## 4. Conclusions

*Cedecea* species are members of the *Enterobacteriaceae* family that have been found in a wide range of natural environments, as well as in human clinical specimens. Reported clinical isolates have been associated with a spectrum of acute infections (e.g., pneumonia, bacteremia, oral ulcers, and dialysis-related peritonitis) in primarily immunocompromised hosts, and antibiotic susceptibility testing has indicated varying degrees of drug resistance among documented isolates. As emerging opportunistic pathogens of environmental origin, *C. neteri* and the closely related species *C. davisae* and *C. lapagei* have received little research attention to date. This study exploited whole-genome sequence information for three *C. neteri* strains (SSMD04, M006, and ND14a) to gain a deeper understanding of the genetic potential for drug resistance in this species and to identify drug-resistance candidate genes for further investigation. We focused our genomic analyses on *C. neteri* SSMD04, an isolate originating from retailed sashimi, since only a draft genome was available for type strain *C. neteri* ATCC 33855. Our work reports the presence of multiple β-lactamase-encoding genes in the *C. neteri* SSMD04 chromosome, including four putative MBLs, a CMY/ACT-type AmpC variant, and a novel β-lactamase gene not described previously. Homologous genes encoding RND- and MFS-type efflux pumps were also identified, along with associated regulatory genes known to be involved in the control of these efflux systems in other bacteria. Comparative analysis of predicted genomic islands suggested the acquisition of some drug resistance determinants and virulence factors by horizontal genetic transfer. The findings of this study advance our currently limited understanding of the molecular basis of antimicrobial resistance in *C. neteri*. Future research is needed to correlate the genetic data with resistance phenotypes impacting public health management of this opportunistic pathogen.

## Figures and Tables

**Table 1:** Completely sequenced genomes of bacterial strains analyzed in this study.

Strain	Chromosome Size (bp)	Protein-Coding Genes	Source	Reference
*Cedecea neteri* SSMD04	4,876,443	4318	Pickled mackerel sashimi	[27]
*Cedecea neteri* M006	4,965,436	4423	Malaysian waterfall	[28]
*Cedecea neteri* ND14a	4,659,311	4141	Malaysian waterfall	Unpublished
*Klebsiella michiganensis* RC10	5,107,633	4536	Rice field	Unpublished

**Table 3:** Annotated genes in the predicted genomic island GI-1 of *C. neteri* and *K. michiganensis*.

*C. neteri* SSMD04			*K. michiganensis* RC10		
Gene Locus	GenBank Accession No.	Gene Product	Gene Locus	GenBank Accession No.	Gene Product
JT31_RS16805	WP_038479661.1	Transposase	VW41_RS18405	WP_045783700.1	Outer membrane protein assembly factor BamE
JT31_RS16810	None	Integrase	VW41_RS18410	WP_045783701.1	RnfH family protein
JT31_RS16815	WP_038479664.1	Secretion protein HlyD	VW41_RS18415	WP_045783702.1	Ubiquinone-binding protein
JT31_RS16820	WP_038479667.1	ATP-binding protein	VW41_RS18420	WP_008458185.1	SsrA-binding protein
JT31_RS16825	WP_038479668.1	Type I secretion protein TolC	VW41_RS18425	WP_045783703.1	Large repetitive protein
JT31_RS16830	WP_038479669.1	Large repetitive protein	VW41_RS18430	WP_045783704.1	Type I secretion protein TolC
JT31_RS16835	WP_038479670.1	SsrA-binding protein	VW41_RS18435	WP_045783705.1	ATP-binding protein
JT31_RS16840	WP_038479681.1	Ubiquinone-binding protein	VW41_RS18440	WP_045783706.1	HlyD family type I secretion periplasmic adaptor subunit
JT31_RS16845	WP_038479684.1	RnfH family protein	VW41_RS18445	WP_045783707.1	Integrase
JT31_RS16850	WP_038479687.1	Outer membrane protein assembly factor BamE	VW41_RS18450	WP_045783708.1	ATP-dependent DNA helicase
JT31_RS23250	WP_071842976.1	Hypothetical protein	VW41_RS18455	WP_052699081.1	Chromosome segregation protein SMC
JT31_RS16855	WP_038479690.1	DNA repair protein RecN	VW41_RS18460	WP_045783709.1	Hypothetical protein
			VW41_RS18465	WP_045783710.1	Hypothetical protein
			VW41_RS18470	WP_045783711.1	Hypothetical protein
			VW41_RS18475	WP_045783712.1	Hypothetical protein
			VW41_RS24485	None	Relaxase
			VW41_RS18480	WP_045783713.1	Transposase
			VW41_RS18485	WP_071844405.1	Transposase
			VW41_RS18490	WP_045783715.1	Hypothetical protein
			VW41_RS18495	WP_045783716.1	Hypothetical protein

## Data Availability

Not applicable.
